# Highly durable and flexible gallium-based oxide conductive-bridging random access memory

**DOI:** 10.1038/s41598-019-50816-7

**Published:** 2019-10-02

**Authors:** Kai-Jhih Gan, Po-Tsun Liu, Ta-Chun Chien, Dun-Bao Ruan, Simon M. Sze

**Affiliations:** 1 Department of Electronics Engineering, National Chiao Tung University, Hsinchu, 30010 Taiwan; 2 Department of Photonics and Institute of Electro-Optical Engineering, National Chiao Tung University, Hsinchu, 30010 Taiwan

**Keywords:** Electronic devices, Information storage, Electronic and spintronic devices

## Abstract

The flexible conductive-bridging random access memory (CBRAM) device using a Cu/TiW/Ga_2_O_3_/Pt stack is fabricated on polyimide substrate with low thermal budget process. The CBRAM devices exhibit good memory-resistance characteristics, such as good memory window (>10^5^), low operation voltage, high endurance (>1.4 × 10^2^ cycles), and large retention memory window (>10^5^). The temperature coefficient of resistance in the filament confirms that the conduction mechanism observed in the Ga_2_O_3_ layer is similar with the phenomenon of electrochemical metallization (ECM). Moreover, the performance of CBRAM device will not be impacted during the flexibility test. Considering the excellent performance of the CBRAM device fabricated by low-temperature process, it may provide a promising potential for the applications of flexible integrated electronic circuits.

## Introduction

Flexible electronics are critical technologies for the development of wearable electronic equipment so that various electronic components, such as, organic light-emitting diodes (OLEDs), solar cells, sensors, and thin film transistors (TFTs), have been widely demonstrated on flexible substrates by many researchers^1–9^. Based on the trend, the demand for the flexible memory devices will also increase to assist the growth of flexible electronics. However, the conventional Flash memory is difficult to be integrated into flexible substrates due to the gate oxide quality degradation for the limited low-temperature process^10,11^. Notably, nonvolatile resistive random access memory (RRAM) was proposed and is the most promising candidate because of its simple structure, low temperature process, high scalability, and high packaging density^12–14^. A large amount of metal oxides have been studied for the RRAM applications^15–18^. Among of them, gallium oxide (Ga_2_O_3_) is well-known for its wide bandgap (E_g_ ~ 4.9 eV), high dielectric constant, and compatible fabrication since it can be fabricated at room temperature^19–21^. In addition, gallium (Ga) is commonly used in the display industry to control the oxygen vacancy in the InGaZnO material due to its ease of combination with oxygen ions^22^. Therefore, Ga_2_O_3_ can be considered as one of the promising candidates for RRAM devices. The widely recognized physical mechanism in RRAM devices can be divided into two categories, one is the oxide resistive random access memory (OxRRAM) and the other is the conductive-bridging random access memory (CBRAM)^23,24^. For the OxRRAM, the formation and rupture of the filaments is formed by oxygen vacancies within the RRAM device^25–27^. CBRAM is also referred to as electrochemical metallization (ECM) memory, relying on the formation/dissolution of metallic filaments inside the switching layer^28–31^. CBRAM shows larger memory window and more power usage effectiveness compared to the OxRAM^32,33^. The Cu-based CBRAM, which takes the advantage of the Cu line as the top electrode, is also a beneficial choice with respect to the reduction of RC propagation delay and cost-effective fabrication^34,35^. Flexible CBRAM will play an increasingly important role in flexible electronic systems, including data processing, information storage, and communication with external devices.

In this work, the device consisted of stacked Cu/TiW/Ga_2_O_3_/Pt structure is demonstrated on low-cost polyimide (PI) substrate processed at low temperature. Electrical characteristics of Ga_2_O_3_ CBRAM devices are discussed in details, such as set/reset voltage, DC endurance property, and data retention. Good memory window with large high to low-resistance state (HRS/LRS) ratio of 10^5^ can be achieved. In addition, the nature of conductive filament in the Ga_2_O_3_ device is also explored. Highly durable Ga_2_O_3_ CBRAM characteristics are exhibited in this work and provide a stage for the development of wearable electronic technology and large-area electronics.

## Results

**Figure 1:**
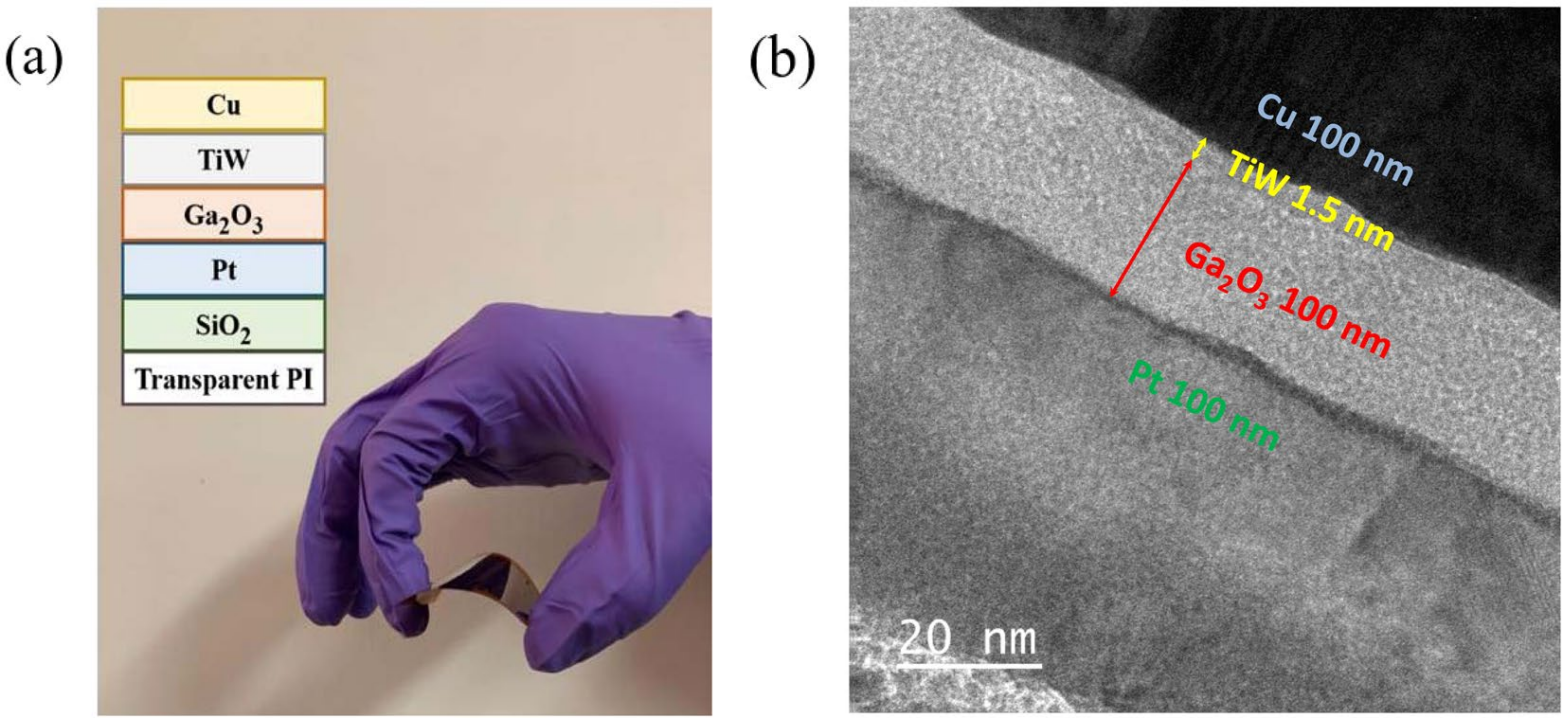
(**a**) Device photo of fabricated CBRAMs on flexible PI substrate. (**b**) Cross-sectional TEM image of the Cu/TiW/Ga_2_O_3_/Pt with 200 °C annealing in N_2_ atmosphere.

Figure 1(a) shows the devices structure of Cu/TiW/Ga_2_O_3_/Pt CBRAM fabricated on flexible PI substrate. A TEM image of Ga_2_O_3_ CBRAM device annealed at 200 °C in N_2_ atmosphere clearly indicates that the thicknesses of the bottom electrode (Pt), switching layer (Ga_2_O_3_), barrier layer (TiW), and top electrode (Cu) layer are 100, 1.5, 20, and 100 nm, respectively.

**Figure 2:**
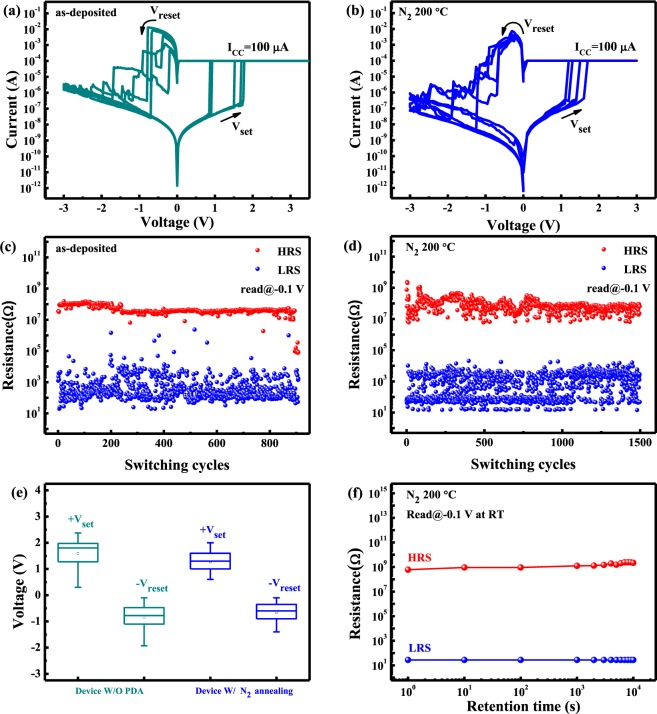
The typical bipolar I-V switching curves of (**a**) Ga_2_O_3_ flexible CBRAM devices and (**b**) Ga_2_O_3_ flexible CBRAM devices with 200 °C annealing in N_2_ atmosphere. The DC endurance characteristics of (**c**) as-deposited and (**d**) 200 °C N_2_ annealing Ga_2_O_3_ flexible CBRAM devices (**e**) The statistical distribution of V_set_ and V_reset_ in the as-deposited and 200 °C N_2_ annealing Ga_2_O_3_ flexible CBRAM devices. (**f**) Retention of 200 °C N_2_ annealing Ga_2_O_3_ flexible CBRAM devices at room temperature.

For both forming and set processes, the current compliance of 100 μA is crucial to avoid a permanent or hard breakdown. The forming process was required to activate the CBRAM devices under DC sweeping mode. The forming voltage of the as-deposited Ga_2_O_3_ CBRAM device was 5.9 V. However, the forming voltages of the device annealed at 200 °C in N_2_ atmosphere is significantly decreased to 4.4 V. Figure 2(a,b) show the typical bipolar current-voltage (*I-V*) curves of the as-deposited CBRAM device and the device annealed at 200 °C on flexible PI, respectively. In the beginning, the low-resistance state (LRS) is achieved when conductive filaments in the Ga_2_O_3_ switching layer are produced by Cu ion migration and stacking from the Cu to Pt bottom electrode in the Ga_2_O_3_ layer; it’s called the set process. Conversely, the high-resistance state (HRS) is achieved when conductive filaments in the switching layer are ruptured by Cu ion migration and stacking from the Pt to Cu electrodes in the Ga_2_O_3_ layer; this is called the reset process. The DC endurance of the as-deposited device and the device annealed at 200 °C in N_2_ atmosphere are depicted in Fig. 2(c,d), respectively. The performance of the as-deposited device is stable only up to 700 cycling endurance, while the device annealed at 200 °C in N_2_ atmosphere exhibits the better endurance cycles over 1.4 × 10^3^. In order to investigate the uniformity of the switching parameters, the statistical variations in set voltage (V_set_) and reset voltage (V_reset_) for both devices were checked. Figure 2(e) indicates a substantial difference in the statistical distributions of V_set_ and V_reset_ between the devices. The coefficient of variation (CV) expresses the variation as a percentage of the mean, and is defined as the ratio of the standard deviation (σ) to the mean value (μ). The distribution is improved from the as-deposited devices (CV_set_ = 33.8%, CV_reset_ = 58.8%) to the device annealed at 200 °C in N_2_ atmosphere (CV_set_ = 29.1%, CV_reset_ = 57.8%). Moreover, the retention characteristics of our device are also studied, as shown in Fig. 2(f). For Ga_2_O_3_ device annealed at 200 °C in N_2_ atmosphere, HRS and LRS are quite stable, without significant resistance decay (HRS/LRS > 10^5^) even after 10^4^ s operation at room temperature. According to the results mentioned above, the CBRAM annealed at 200 °C in N_2_ atmosphere shows good performance and evinces its potential for the memory applications.

**Figure 3:**
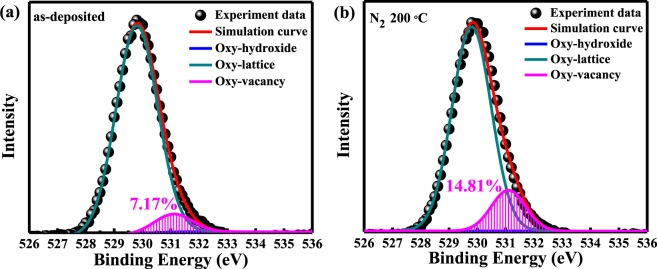
Analysis of the XPS O 1 s spectrum on (**a**) the as-deposited Ga_2_O_3_ thin film and (**b**) and Ga_2_O_3_ thin film annealed at 200 °C in N_2_ atmosphere.

To further investigate the physical mechanism for the endurance improvement caused by the post-deposition annealing in N_2_ atmosphere, material analysis was performed. The XPS spectra were performed with PHI Quantera SXM, using Al Kα source (a beam power of 25 W and an emission current of 4.025 mA). The XPS spectrum of the O 1 s signal was applied to examine the oxygen binding states at the surface of the Ga_2_O_3_ thin film. The XPS result of the sample with Ga_2_O_3_ switching layer without annealing process is shown in Fig. 3(a). The O 1 s peak can be fitted by two nearly Gaussian distribution peaks, approximately located at 529.8 (FWHM = 1.6) and 531.1 eV (FWHM = 1.5), respectively. The lower binding energy peak located at 529.8 eV is attributed to oxygen-lattice bonds, which are related to the O^2−^ ions combined with the Ga atoms in the Ga_2_O_3_ compound system. On the other hand, the higher binding energy peak located at 531.1 eV can be attributed to oxygen-vacancy (O_V_) bonds. The O_V_ peak is attributed to the oxygen deficient in the Ga_2_O_3_ matrix. According to the XPS results shown in Fig. 3(a,b), it is important to notice that the proportion of oxygen-vacancy bonds (14.81%) of Ga_2_O_3_ film annealed at 200 °C in N_2_ atmosphere is higher than the one without annealing (7.17%). Therefore, the Ga_2_O_3_ CBRRAM devices at 200 °C in N_2_ atmosphere exhibit good switching behaviors and lower V_set_ and V_reset_ caused by a considerable amount of oxygen vacancies in the oxide layer. This improvement can be attributed to these considerable oxygen vacancies which may lower the energy cost of Cu insertion into the Ga_2_O_3_ layer, and lead to the Cu migration in the switching layer^32^.

**Figure 4:**
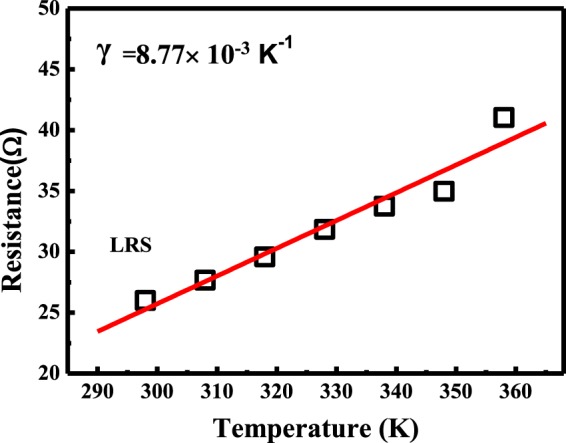
Temperature-dependent LRS resistance of Ga_2_O_3_ CBRAM devices; the solid lines are the linear fit with an equation R(T) = R(T_o_) [1 + γ(T − T_o_)].

In order to further understand the nature of conductive filaments, the conduction mechanism can be confirmed by the curve fitting. Firstly, we consider a linear temperature dependence of the conductive filament typical of metallic behavior, that is, R(T) = R(T_0_) [1 + γ(T − T_0_)], where R(T) is the LRS resistance at temperature T, R(T_0_) is the LRS resistance at room temperature T_0_, and γ is the temperature coefficients of resistance^36,37^. The LRS resistance of the devices is taken from 298 to 358 K and linearly increases with temperature, as shown in Fig. 4. For our devices, the temperature coefficients of resistance (γ) is about 8.77 × 10^−3^ K^−1^. As shown in Table 1, the result is one order of magnitude more than that of oxygen vacancy assisted filaments (6.03 × 10^−4^ K^−1^)^38^. The temperature coefficients obtained are close to the value 1.3 × 10^−2^ K^−1^ for high-purity Cu assisted filaments^39^. Therefore, the formation and rupture of Cu-based conductive filaments is responsible for the resistive switching behavior in our devices.

**Table 1:** Comparison of the resistance temperature coefficient (γ) of different RRAM devices.

RRAM	Cu/TiW/ZrO_2_/TiN CBRAM^39^	Ga_2_O_3_ CBRAM (this work)	Cu/Ta_2_O_5_/TiN CBRAM^37^	HfOx OxRRAM^40^	ZnO OxRRAM^38^
γ(K^−1^)	1.3 × 10^−2^	8.77 × 10^−3^	3 × 10^−3^	8 × 10^−4^	6.03 × 10^−4^
Types of conductive filament	Cu	Cu	Cu	Oxygen vacancy	Oxygen vacancy

**Figure 5:**
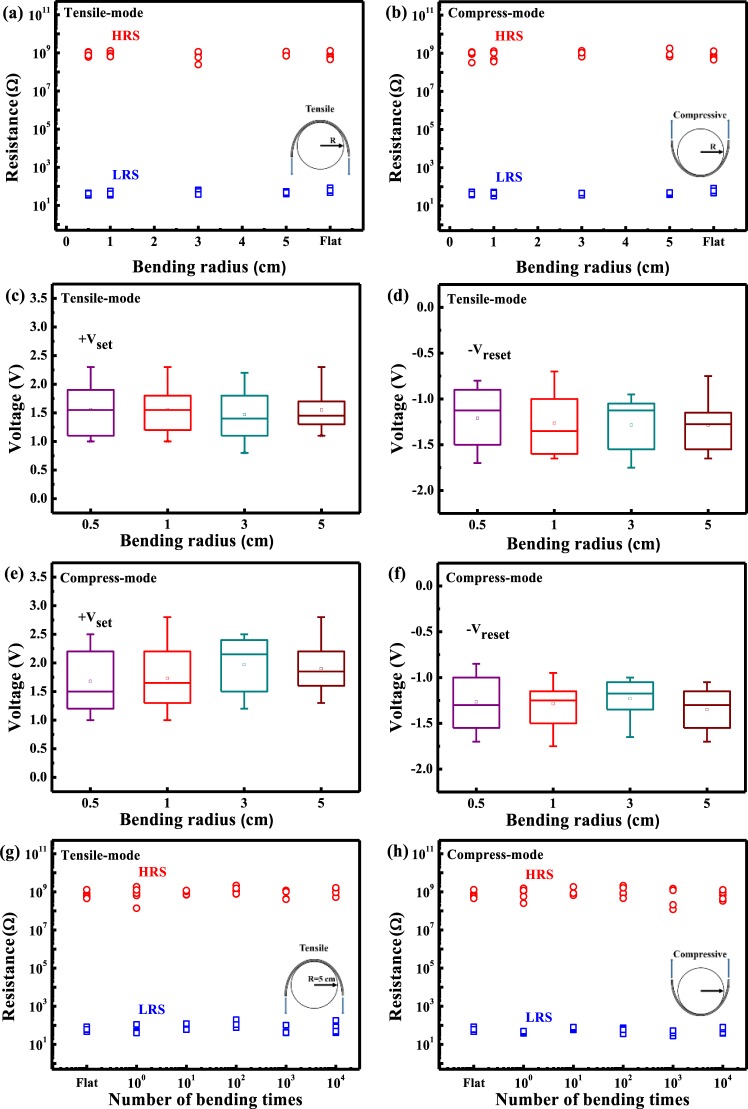
The values of LRS/HRS resistances after the device underwent different bending radius at (**a**) tensile test and (**b**) compressive test. (**c**,**d**) Statistical distributions of V_set_ and V_reset_ after the device underwent different bending radius at the tensile test. (**e**,**f**) Statistical distributions of V_set_ and V_reset_ after the device underwent different bending radius at the compressive test. Comparison of the current ratio between the LRS and HRS states of the flat and bent devices, along with the device after 10^4^ continuous bending cycles at (**g**) tensile test and (**h**) compressive test. All data; five different devices were measured each time.

Good mechanical flexibility is crucial for applications in flexible electronics. Then, the substrate is bent to different curvature radius of 5.0, 3.0, 1.0, and 0.5 cm with both tensile and compressive stresses. After each bending operation, the performance is evaluated by recording I-V curves on five different devices. As shown in Fig. 5(a,b), the R_LRS_/R_HRS_ values are stable at more than 10^5^ even after it is bent with different curvature radius. The statistical data in Fig. 5(c–f) may reveal that V_set_, and V_reset_ can be kept, almost the same with those of a fresh device. Furthermore, a continuous bending test of up to 10^4^ times with curvature radius of 5.0 cm is carried out. Even at 10^4^ bends, the R_LRS_/R_HRS_ can be maintained, as shown in Fig. 5(g,h). These results indicate that a flexible Ga_2_O_3_ CBRAM device with 200 °C annealing in N_2_ atmosphere exhibits good flexibility, which suggests its possibility of commercially viable nonvolatile memory devices.

In summary, an amorphous Ga_2_O_3_ CBRAM device annealed at 200 °C in N_2_ atmosphere on low-cost flexible substrate is proposed for achieving good memory window, stable retention characteristics and a 2 times enhancement in endurance in this study. Through the current-voltage measurements, the device can be reliably operated in a low set voltage of 1.3 V and a low reset voltage of −0.65 V. The mechanism of the endurance enhancement effects is also ascribed to the generation of a considerable amount of oxygen vacancies in the Ga_2_O_3_ layer by N_2_ annealing, which may lead to the formation of Cu filaments. Analysis of the XPS O 1 s depth distribution profile has been used to confirm this inference. Therefore, oxygen vacancies play a crucial role in resistive switching mechanism in Ga_2_O_3_ CBRAM device. The extraction about the temperature dependence of resistance is an effectual method to examine the nature of the conductive filaments. The temperature coefficient of resistance is about 8.77 × 10^−3^ K^−1^ in this work, which means the devices are ECM-based RRAM with Cu filament. These results indicate that the flexible Ga_2_O_3_ CBRAM is attractive for low-cost wearable devices and suitable for the future bendable displays.

## Methods

The CBRAM using Cu/TiW/Ga_2_O_3_/Pt stacks were successfully fabricated with limited process temperature of 200 °C on a flexible PI substrate. Firstly, the PI substrate was cleaned ultrasonically with ethanol for 5 mins and DI water. After cleaning up a PI substrate, a thin 100 nm SiO_2_ buffer layer was deposited on PI substrate by plasma enhanced chemical vapor deposition. Then, 100 nm-thick Pt bottom electrode with 5 nm-thick Ti adhesion layer was deposited by using direct-current (DC) magnetron sputtering. Then, a 20 nm-thick Ga_2_O_3_ film was deposited on a Pt/Ti/SiO_2_/PI substrate by radio-frequency magnetron sputtering at room temperature. The deposition pressure was kept at 0.4 Pa, while the ratio of Ar:O_2_ gas flow was kept at 1:1. The optimization of Ga_2_O_3_ resistive switching layer deposition was applied in this work. Except for the control sample, a post-deposition annealing in N_2_ atmosphere is applied at 200 °C for 30 mins. Finally, 1.5 nm-thick TiW barrier layer and 100 nm-thick Cu top electrodes were deposited by using DC magnetron sputtering and patterned with a shadow mask with a diameter of 100 μm. Electrical measurements were recorded by Keithley 4200 semiconductor characterization analyzer. To investigate the endurance improvement, transmission electron microscope (TEM) and X-ray photoelectron spectroscopy (XPS) were used to analyze the material properties.
